# Indocyanine green combined with methylene blue versus methylene blue alone for sentinel lymph node biopsy in breast cancer: a retrospective study

**DOI:** 10.1186/s12893-023-02037-z

**Published:** 2023-05-17

**Authors:** Qiu-hui Yang, Xiang-jian Zhang

**Affiliations:** 1 Wenzhou Medical University, Wenzhou, Zhejiang 325035 China; 2 The Dingli Clinical College of Wenzhou Medical University, Wenzhou, Zhejiang 325000 China; 3 Department of Surgical Oncology, Wenzhou Central Hospital, Wenzhou, Zhejiang 325000 China; 4 The Second Affiliated Hospital of Shanghai University, Wenzhou, Zhejiang 325000 China

**Keywords:** Indocyanine green (ICG), Methylene blue (MB), Breast cancer, Sentinel lymph node biopsy (SLNB), Sentinel lymph nodes (SLNs)

## Abstract

**Background:**

Recent studies have shown that near-infrared (NIR) fluorescence imaging using Indocyanine green (ICG) may improve the efficiency of sentinel lymph node biopsy (SLNB). This study aimed to assess the effectiveness of the combination of ICG and methylene blue (MB) in breast cancer patients undergoing SLNB.

**Patients and method:**

We evaluated ICG plus MB (ICG + MB) identification effectiveness with MB alone using retrospective analysis. From 2016 to 2020, we collected data on 300 eligible breast cancer patients who got SLNB treatment in our institution by ICG + MB or MB alone. By comparing the distribution of clinicopathological characteristics, the detection rate of sentinel lymph nodes (SLNs) and metastatic SLNs, as well as the total number of SLNs in the two groups, we were able to assess the imaging efficiency.

**Results:**

Fluorescence imaging allowed 131 out of 136 patients in the ICG + MB group to find SLNs. ICG + MB group and MB group had detection rates of 98.5% and 91.5% (*P* = 0.007, χ^2^ = 7.352), respectively. Besides, the ICG + MB approach was able to produce improved recognition outcomes. What’s more, compared with the MB group, the ICG + MB group can identify more lymph nodes (LNs) (3.1 to 2.6, *P* = 0.000, t = 4.447). Additionally, in the ICG + MB group, ICG could identify more LNs than MB (3.1 vs 2.6,* P* = 0.004, t = 2.884).

**Conclusion:**

ICG has high detection effectiveness for SLNs, and when paired with MB, the detection efficiency can be increased even further. Furthermore, the ICG + MB tracing mode does not involve radioisotopes, which has a lot of promise for clinical use and can take the place of conventional standard detection methods.

## Background

The condition of axillary lymph nodes is essential for the treatment and prognosis of breast cancer patients [1]. Sentinel lymph node biopsy (SLNB) is widely used to evaluate axillary lymph node staging, which is considered a standard treatment method for patients with early breast cancer without axillary lymph node metastasis [2].

At present, the commonly used method for finding sentinel lymph nodes (SLNs) is a combination of radionuclide and blue dye [3]. The radionuclide approach has the benefits of precise location and simplicity of use, however, it requires specialized detecting equipment and is costly. Importantly, it also has disadvantages like radioactive pollution. These flaws compel us to search for effective tracers devoid of radioactivity. Methylene blue (MB) can be used as a tracer to detect SLNs, because it has a high identification rate, specificity, and sensitivity according to many studies in China [4]. As a result, numerous institutions in developing countries started performing SLNB using MB alone. The MB approach offers the benefits of low-cost and simple preoperative preparation while also protecting clinicians and patients from radiation damage. However, disadvantages such as a high leakage rate, long visualization time, and difficult incision selection may have a negative impact on the patient's prognosis [5, 6].

In recent years, ICG has been used for breast cancer patients who underwent SLNB, which is reported to be a highly sensitive method [6]. In this method, ICG is injected subcutaneously, and an excitation lighting system and a highly sensitive camera are used to track the process of ICG reaching SLNs through lymphatic vessels in real-time. Due to the principle of near-infrared fluorescence imaging being similar to that of the radionuclide method, the combination of ICG and MB (ICG + MB) has great potential for clinical application and is expected to replace the dual tracer method of radionuclide and dye [6]. ICG + MB in SLNB of early breast cancer has been routinely carried out in our hospital. Therefore, we retrospectively analyzed the detection rate and number of SLNs in patients with ICG + MB with MB alone, and the clinicopathological factors were also analyzed to evaluate whether ICG + MB can improve the performance of SLNs tracking in patients with early breast cancer.

## Materials and methods

### Inclusion criteria

We included the data of 300 breast cancer patients who received surgical treatment in Wenzhou Central Hospital from March 2016 to March 2020. These patients' postoperative pathological staging ranged from T1N0M0 to T2N2M0 (Judging criteria according to CSCO guidelines). However, patients with secondary surgery, huge tumor, incomplete imaging, radiotherapy or chemotherapy, and distant metastasis were excluded.

### Materials

SLNs tracer uses 2 ml MB injection with a concentration of 1% (Jichuan Pharmaceutical Group Co., Ltd.) and 1 ml ICG solution with a concentration of 1.25% (Dandong Yichuang Pharmaceutical Co., Ltd.). Fluorescence detector uses a fluorescent vascular imager (Mingde Pharmaceutical Co., Ltd).

### Methods

#### MB staining

Inject 2 ml MB injection subcutaneously into the affected breast areola and massage for 5 min. Select the incision to parallel to the axillary fold wall, and remove the blue-stained SLNs under the guidance of MB.

#### ICG + MB method

**Fig. 1:**
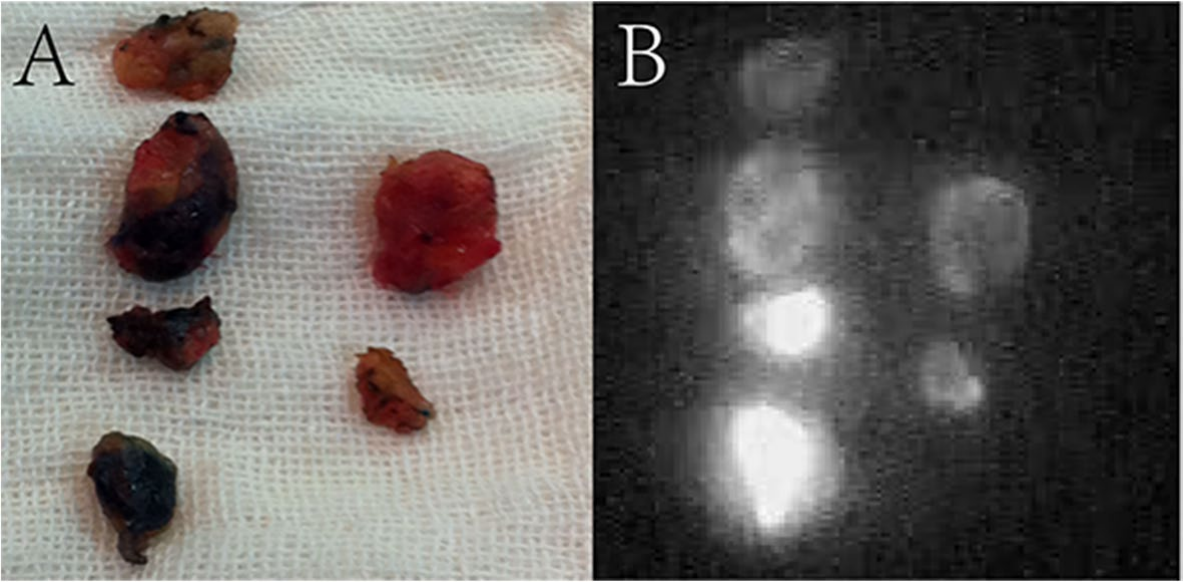
SLNs development status under ICG + MB **A** The development of SLNs observed by the naked eye; **B** The development state of the same SLNs on the left side under the fluorescence instrument, and the intraoperative records were as follows: ①B/G, ②B/G, ③B/G, ④B/G, ⑤G, ⑥G。

Three min after injection of 1 ml MB (the same method as above), observe the fluorescence development immediately after injection of 1 ml ICG again, 2 min after ICG injection, the skin incision was made according to the direction of lymphatic vessels, and the blue stained and fluorescent SLNs were removed together. The removed nodules were divided into three groups: B (MB staining), G (fluorescent imaging), and B/G (MB staining and fluorescent imaging), as shown in Fig. 1.

##### Statistical methods

SPSS25.0 statistical software was used to analyze the data. The measurement data are expressed by mean ± standard deviation (x ± s), using a t-test; And the counting data are expressed by rate (%), using χ^2^ test. The difference was statistically significant (*P* < 0.05).

## Result

### General information

The general data of patients are shown in Table 1. In the ICG + MB group, the lesions of 75 patients were located in the left breast (55.1%). The tumor of 48 patients was located in the Upper outer quadrant (35.3%), the diameter (d) of the focus in most patients does not exceed two centimeters (cm) (75.7%), and the body mass index (BMI) of 89 patients was within the normal range (65.4%). Additionally, a total of 164 patients were traced with MB, the tumor of 75 patients were located in the left breast (53.0%), 61 patients were located in the Upper outer quadrant (37.2%), most of the tumors were d ≤ 2 cm (68.3%), and 99 patients had a BMI within the normal range (60.4%). There was no significant difference between the ICG + MB method and the MB staining method in tumor site, tumor distribution, tumor size, and BMI.

**Table 1:** Clinical data of breast cancer patients

	ICG + MB (*n* = 136)		MB (*n* = 164)		*P* value
	*N*	*%*	*N*	*%*	
**Tumor site**					0.43
Left breast	75	55.1	87	53.0	
Right breast	61	44.9	77	47.0	
**Tumor distribution**					0.67
Upper outer	48	35.3	61	37.2	
Lower outer	11	8.1	8	4.9	
Upper inner	35	25.7	42	25.6	
Lower inner	7	5.1	8	4.9	
Posterior nipple	1	0.7	4	2.4	
Right inner side	5	3.7	11	6.7	
Right outer side	11	8.1	18	11	
Directly above	8	5.9	10	6.1	
Directly below	10	7.4	2	1.2	
**Tumor size**					0.15
T1 (0-2 cm)	103	75.7	112	68.3	
T2 (2-5 cm)	33	24.3	52	31.7	
**BMI (Kg/m** ^**2**^ **)**					0.26
< 19	14	10.3	23	14.0	
19–25	89	65.4	99	60.4	
≥ 25	33	24.3	42	25.6	

*ICG* Indocyanine green, *MB* Methylene blue, *T* Tumor, *BMI* Body mass index

### Comparison of detection rate of SLNs between ICG + MB and MB

SLNs were successfully detected in 134 of 136 patients in the ICG + MB group (98.5%, 134/136), while 150 of 164 patients in the MB group (91.5%, 150/164) *P* = 0.007, χ^2^ = 7.352. Among 134 patients who successfully detected SLNs in the ICG + MB group, 20 patients had lymph node metastasis, with a positive rate of 14.7%, however, there was no significant difference between the ICG + MB group and the MB group (*p* = 0.135, χ^2^ = 2.230). The ICG + MB group showed a total of 421 SLNs, with an average of 3.1 ± 0.9. The MB alone showed a total of 425 SLNs, with an average of 2.6 ± 1.1 (*p* = 0.000, t = 4.447). As is shown in Tables 2 and 3.

**Table 2:** SLNs detection results of ICG + MB group and MB group

	ICG + MB (*n* = 136)		MB (*n* = 164)		*P*
	*N*	%	*N*	%	
SLNs	134	98.5	150	91.5	*p* = 0.007(χ^2^ = 7.352)
SLNs ( +)	20	14.7	15	9.1	*p* = 0.135(χ^2^ = 2.230

*ICG* Indocyanine green, *MB* Methylene blue, *N* Numbers, *SLNs* Sentinel lymph nodes

**Table 3:** Comparison of the number of SLN among groups

	ICG + MB (*n* = 136)		MB (*n* = 164)		*P*
	LNs	**Χ ± S**	LNs	**Χ ± S**	
Numbers	421	3.1 ± 0.9	425	2.6 ± 1.1	*P* < 0.005(t = 4.447)

*ICG* Indocyanine green, *MB* Methylene blue, *LNs* Lymph nodes, *Χ*± *S* Mean ± Standard deviation

### Analysis of SLNs in the ICG + MB group

Among 136 breast cancer patients in the ICG + MB group, 2 patients failed to detect SLNs (1.5%). SLNs were successfully detected in 134 patients (98.5%), including 131 patients (96.3%) with ICG imaging; 124 patients (92.5%) were successfully developed with MB. A total of 421 SLNs were detected in the ICG + MB group (mean 3.1 ± 0.9). 412 SLNs were detected in the ICG group (mean 3.0 ± 1.0); There were 359 SLNs developed with MB (mean 2.6 ± 1.2). The number of SLNs detected by the ICG + MB method was higher than that of MB in the group (*P* = 0.001, t = 3.513). The number of SLNs developed by ICG in the group was higher than that developed by MB (P3 = 0.004, t = 2.884). It was also found that there was no significant statistical difference in the number of SLNs developed between the ICG + MB group and the ICG in the group (P2 = 0.561, t = 0.582). Intra-group analysis showed that ICG had a better tracing effect than MB, and when ICG was combined with MB, the development efficiency was increased, although statistically insignificant, this may be related to the small sample size. As is shown in Table 4.

**Table 4:** Staining details of SLNs in the ICG + MB group

*N* = 136	ICG + MB group ^a^(*n* = 134)		MB group ^b^(*n* = 124)		ICG group ^c^(*n* = 131)		*P1*(a vs b)	*P2*(a vs c)	*P3*(b vs c)
	LNs	**Χ ± S**	LNs	**Χ ± S**	LNs	**Χ ± S**			
Numbers	421	3.1 ± 0.9	359	2.6 ± 1.2	412	3.0 ± 1.0	*P1* = 0.001 (t = 3.513)	*P2* = 0.561 (t = 0.582)	*P3* = 0.004 (t = 2.884)

*ICG* Indocyanine green, *MB* Methylene blue, *N* Numbers, *LNs* Lymph nodes (LNs), *Χ*± *S* Mean ± Standard deviation

## Discussion

This is a retrospective study to evaluate whether the addition of ICG to the widely used MB method can improve SLNs recognition in patients with early breast cancer. We concluded that the detection rate of SLNs with the ICG + MB group was significantly higher than that with MB alone (98.5% vs 91.5%). This finding is consistent with earlier research [7, 8].

The ICG + MB mode with a high recognition rate and high accuracy is expected to become a new non-radioactive SLN tracking scheme. The use of alternative radioactive reagents is quite attractive, especially in institutions where radioisotopes are not readily available. Ballardini et al. of the European Institute of Oncology reported a trial that compared the tracking ability of ICG and ^99m^Tc in 134 breast cancer patients. The research results of this equivalent design prove that the detection rate of SLNs using the ICG method is not less than.^99m^Tc [9, 10]. Although Ahmed M. criticized the lack of standard dual mapping technology in Ballardini's research, it still showed hope to replace radioactive tracers with ICG [11–13].

We can conclude from this study that the SLNs detection rate of ICG + MB is 7.0% higher than that of MB, which is equivalent to the difference between the double staining method including standard radioisotopes and the single use of dyes, indicating that ICG has a good tracing effect [14, 15]. Although few studies compare the SLNs recognition performance of ICG with that of the nuclide + dye method, we can indirectly conclude that ICG + MB is the most likely alternative method at present [16, 17].

ICG can not only improve the detection rate of SLNs but also detect more metastatic SLNs [18]. Initially, ICG has an extremely high sensitivity to develop some SLNs undetected by MB. This research result shows that the average number of SLNs detected by ICG + MB is 3.1, which is greater than the average number of SLNs detected by the MB method. Similar to previous studies [19]. We hypothesize that the high visibility of ICG's high-resolution near-infrared equipment is what accounts for its exceptional sensitivity. The results of two meta-analyses showed that detecting more SLNs status (3 to 4 nodes) could better understand the status of axillary lymph nodes and was also related to the prognosis of patients, while only one SLN could not fully represent the axillary status [20, 21].

As previously reported, ICG has several limitations. Firstly, the penetration of NIR fluorescence in tissues is lower than γ radial, which cause a worrying problem [22]. The use of ICG tracing for obese breast cancer patients will lead to the detection failure of SLNs. Although Kitai reported the axillary skin compression technology that has not been used in the current study, there was no significant difference between obese patients (BMI ≥ 25) and non-obese patients [23]. However, Grischke's research result shows that when the patient's BMI is lower than 40, the detection rate of ICG for SLNs has not been affected. Only when the patient's BMI is higher than 40, the detection rate of ICG for SLNs will decline [24]. Few Chinese patients have a BMI > 30, which is consistent with the patient information included in this study. Therefore, in China, low penetration of ICG will not become an obstacle to the clinical application [25]. Secondly, ICG also has the feature of fluorescence quenching. According to relevant reports, with the increase of ICG concentration, the fluorescence signal of ICG will be quenched (the reduction of fluorescence emission). Mieog recommended 0.62 mg as the optimal injection dose of ICG after the study [26]. However, there is no consensus on the optimal dose of ICG. Based on the previous study, we chose 1.25 mg ICG as the injection dose. The excellent navigation performance showed that the dose could be used clinically. Thirdly, allergic reactions, skin damage, and other adverse reactions are the main factors to be considered in the dye method [27, 28]. In this study, we found no cases of allergy or skin damage. Hypodermic injection rather than intravenous injection may be the main reason for less allergic reaction. The main skin complications included temporary skin staining at the injection site, permanent tattoos, and subcutaneous nodules. Although these skin complications may cause a certain degree of anxiety in patients, they are acceptable for most patients. The safety results are similar to those of the previous meta-analysis [29].

The current study has two shortcomings. On the one hand, we did not compare the ICG + MB method with the ^99m^Tc + MB method. However, the excellent performance of the ICG + MB may be indirectly confirmed by the 5% superiority test. Moreover, some studies have confirmed that there is no significant difference between ICG and RI in SLN tracing, as it can be used as an alternative or complementary method to RI methods. It is recommended to use ICG + MB methods in centers that without radioactive material [30]. On the other hand, this is a single-center retrospective study, and the results need to be further confirmed by a single-center or multicenter prospective study. In a word, this research result reveals the high detection rate of ICG and the advantages of combining ICG fluorescence with blue dye in the detection of SLN in early breast cancer.

## Conclusion

Lymph node navigation through ICG fluorescence has a high detection rate of SLNs, which can further improve the positioning performance when combined with blue dye. Besides, this method does not involve radioisotopes, so the new ICG + MB double tracing mode has great potential for clinical application and can replace the traditional standard detection method.

## Data Availability

The results/data/figures in this manuscript have not been published elsewhere, nor are they under consideration (from you or one of your Contributing Authors) by another publisher. The datasets used and analyzed during the current study available from the corresponding author on reasonable request.

## References

[1] Zhang LL, Ma L, Geng CZ, Jia Y, Wang XL, Liu YP (2017). Correlation between extranodal invasion in axillary lymph node metastasis and prognosis of breast cancer patients. Zhonghua Bing Li Xue Za Zhi.

[2] Krag DN, Anderson SJ, Julian TB, Brown AM, Harlow SP, Costantino JP, Ashikaga T, Weaver DL, Mamounas EP, Jalovec LM (2010). Sentinel-lymph-node resection compared with conventional axillary-lymph-node dissection in clinically node-negative patients with breast cancer: overall survival findings from the NSABP B-32 randomised phase 3 trial. Lancet Oncol.

[3] Giammarile F, Alazraki N, Aarsvold JN, Audisio RA, Glass E, Grant SF, Kunikowska J, Leidenius M, Moncayo VM, Uren RF (2013). The EANM and SNMMI practice guideline for lymphoscintigraphy and sentinel node localization in breast cancer. Eur J Nucl Med Mol Imaging.

[4] Yang S, Xiang HY, Xin L, Zhang H, Zhang S, Cheng YJ, Liu Q, Xu L, Li T, Duan XN (2021). Retrospective analysis of sentinel lymph node biopsy using methylene blue dye for early breast cancer. Chin Med J (Engl).

[5] OʼReilly  EA, Prichard  RS, Al Azawi  D, Aucharaz N, Kelly G, Evoy D, Geraghty J, Rothwell J, OʼDoherty A, Quinn C (2015). The value of isosulfan blue dye in addition to isotope scanning in the identification of the sentinel lymph node in breast cancer patients with a positive lymphoscintigraphy: a randomized controlled trial (ISRCTN98849733). Ann Surg.

[6] Ersoy YE, Kadioglu H (2018). Review of novel sentinel lymph node biopsy techniques in breast cancer patients treated with neoadjuvant chemotherapy. Clin Breast Cancer.

[7] Pitsinis V, Wishart GC (2017). Comparison of indocyanine green fluorescence and blue dye methods in detection of sentinel lymph nodes in early-stage breast cancer. Ann Surg Oncol.

[8] Jin Y, Yuan L, Zhang Y, Tang P, Yang Y, Fan L, Chen L, Qi X, Jiang J (2022). A prospective self-controlled study of indocyanine green, radioisotope, and methylene blue for combined imaging of axillary sentinel lymph nodes in breast cancer. Front Oncol.

[9] Vermersch C, Raia-Barjat T, Chapelle C, Lima S, Chauleur C (2019). Randomized comparison between indocyanine green fluorescence plus (99m)technetium and (99m)technetium alone methods for sentinel lymph node biopsy in breast cancer. Sci Rep.

[10] Dell'Oglio P, de Vries HM, Mazzone E, KleinJan GH, Donswijk ML, van der Poel HG, Horenblas S, van Leeuwen FWB, Brouwer OR (2020). Hybrid Indocyanine Green-(99m)Tc-nanocolloid for single-photon emission computed tomography and combined radio- and fluorescence-guided sentinel node biopsy in penile cancer: results of 740 inguinal basins assessed at a single institution. Eur Urol.

[11] Ahmed M, Douek M (2014). What is the clinical relevance of discordance between radioisotope alone and indocynanine green in sentinel lymph node biopsy for breast cancer?. Eur J Surg Oncol.

[12] Sugie T, Ikeda T, Kawaguchi A, Shimizu A, Toi M (2017). Sentinel lymph node biopsy using indocyanine green fluorescence in early-stage breast cancer: a meta-analysis. Int J Clin Oncol.

[13] Prader S, du Bois A, Harter P, Breit E, Schneider S, Baert T, Heitz F, Traut A, Ehmann S, Pauly N (2020). Sentinel lymph node mapping with fluorescent and radioactive tracers in vulvar cancer patients. Arch Gynecol Obstet.

[14] Manca G, Rubello D, Tardelli E, Giammarile F, Mazzarri S, Boni G, Chondrogiannis S, Marzola MC, Chiacchio S, Ghilli M (2016). Sentinel lymph node biopsy in breast cancer: indications, contraindications, and controversies. Clin Nucl Med.

[15] Valente SA, Al-Hilli Z, Radford DM, Yanda C, Tu C, Grobmyer SR (2019). Near infrared fluorescent lymph node mapping with indocyanine green in breast cancer patients: a prospective trial. J Am Coll Surg.

[16] Thongvitokomarn S, Polchai N (2020). Indocyanine green fluorescence versus blue dye or radioisotope regarding detection rate of sentinel lymph node biopsy and nodes removed in breast cancer: a systematic review and meta-analysis. Asian Pac J Cancer Prev.

[17] Agrawal SK, Hashlamoun I, Karki B, Sharma A, Arun I, Ahmed R (2020). Diagnostic performance of indocyanine green plus methylene blue versus radioisotope plus methylene blue dye method for sentinel lymph node biopsy in node-negative early breast cancer. JCO Glob Oncol.

[18] Lin J, Lin LS, Chen DR, Lin KJ, Wang YF, Chang YJ (2020). Indocyanine green fluorescence method for sentinel lymph node biopsy in breast cancer. Asian J Surg.

[19] Guo J, Yang H, Wang S, Cao Y, Liu M, Xie F, Liu P, Zhou B, Tong F, Cheng L (2017). Comparison of sentinel lymph node biopsy guided by indocyanine green, blue dye, and their combination in breast cancer patients: a prospective cohort study. World J Surg Oncol.

[20] Jaffer S, Bleiweiss IJ (2014). Evolution of sentinel lymph node biopsy in breast cancer, in and out of vogue?. Adv Anat Pathol.

[21] Li X, Chen S, Duan Y, Guo H, Jiang L, Kong X, Ma T, Yang Q (2020). Identification and preservation of stained non-sentinel lymph nodes in breast cancer. Oncol Lett.

[22] Majlesara A, Golriz M, Hafezi M, Saffari A, Stenau E, Maier-Hein L, Müller-Stich BP, Mehrabi A (2017). Indocyanine green fluorescence imaging in hepatobiliary surgery. Photodiagnosis Photodyn Ther.

[23] Kitai T, Kawashima M (2012). Transcutaneous detection and direct approach to the sentinel node using axillary compression technique in ICG fluorescence-navigated sentinel node biopsy for breast cancer. Breast Cancer.

[24] Grischke EM, Röhm C, Hahn M, Helms G, Brucker S, Wallwiener D (2015). ICG fluorescence technique for the detection of sentinel lymph nodes in breast cancer: results of a prospective open-label clinical trial. Geburtshilfe Frauenheilkd.

[25] Wang Y, Xue H, Sun M, Zhu X, Zhao L, Yang Y (2019). Prevention and control of obesity in China. Lancet Glob Health.

[26] Mieog JS, Troyan SL, Hutteman M, Donohoe KJ, van der Vorst JR, Stockdale A, Liefers GJ, Choi HS, Gibbs-Strauss SL, Putter H (2011). Toward optimization of imaging system and lymphatic tracer for near-infrared fluorescent sentinel lymph node mapping in breast cancer. Ann Surg Oncol.

[27] Volders JH, van la Parra RF, Bavelaar-Croon CD, Barneveld PC, Ernst MF, Bosscha K, De Roos WK (2014). Discordance between number of scintigraphic and perioperatively identified sentinel lymph nodes and axillary tumour recurrence. Breast.

[28] Winter A, Kowald T, Paulo TS, Goos P, Engels S, Gerullis H, Schiffmann J, Chavan A, Wawroschek F (2018). Magnetic resonance sentinel lymph node imaging and magnetometer-guided intraoperative detection in prostate cancer using superparamagnetic iron oxide nanoparticles. Int J Nanomedicine.

[29] Choi J, Shin JG, Tak YO, Seo Y, Eom J. Single camera-based dual-channel near-infrared fluorescence imaging system. Sensors (Basel). 2022;22(24):9798. 10.3390/s22249758 PMC9786791 36560127

[30] Goonawardena J, Yong C, Law M (2020). Use of indocyanine green fluorescence compared to radioisotope for sentinel lymph node biopsy in early-stage breast cancer: systematic review and meta-analysis. Am J Surg.

